# Differences in the Regulation of Ochratoxin A by the HOG Pathway in *Penicillium* and *Aspergillus* in Response to High Osmolar Environments

**DOI:** 10.3390/toxins5071282

**Published:** 2013-07-19

**Authors:** Dominic Stoll, Markus Schmidt-Heydt, Rolf Geisen

**Affiliations:** Department of Safety and Quality of Fruits and Vegetables, Max Rubner-Institut, Haid-und-Neu-Str. 9, Karlsruhe 76121, Germany; E-Mails: dominic.stoll@mri.bund.de (D.S.); markus.schmidt-heydt@mri.bund.de (M.S.-H.)

**Keywords:** *Penicillium nordicum*, *Penicillium verrucosum*, *Aspergillus carbonarius*, ochratoxin, osmotic stress, HOG signaling cascade

## Abstract

*Penicillium verrucosum*, *P. nordicum* and *Aspergillus carbonarius* are three important ochratoxin A producing species. *P. verrucosum* is in addition able to produce citrinin. It has been shown earlier that *P. nordicum* is adapted to NaCl rich environments like salt rich dry cured foods or even salines. In this organism, the biosynthesis of ochratoxin A plays an adaptive role in this habitat. *P. verrucosum* generally can be found on cereals, but occasionally also on salt rich dry cured foods. In contrast *A. carbonarius* usually cannot be found in NaCl rich environments, but it occurs in another environment with high concentration of solutes, e.g., in sugar rich substrates like grapes and grape juices. Usually osmotic challenging conditions activate the HOG MAP kinase signal cascade, which in turn activates various osmo-regulated genes. In the current analysis, it could be demonstrated that in case of *P. nordicum* and *P. verrucosum* the NaCl induced production of ochratoxin A is correlated to the phosphorylation status of the HOG MAP kinase. Just the opposite was true for *A. carbonarius*. In this case, also higher amounts of NaCl in the medium lead to an increased phosphorylation status of HOG, but no increase in ochratoxin biosynthesis was observed. In contrast to the Penicillia, higher NaCl concentrations lead to a rapid cessation of growth by *A. carbonarius.* High glucose concentrations have much less impact on growth and the phosphorylation of HOG.

## 1. Introduction

Ochratoxin A is a nephrotoxic mycotoxin produced by some *Aspergillus* and *Penicillium* species. Because of the temperature and humidity preferences ochratoxigenic Aspergilli occur mainly in regions with elevated temperatures and occur on food commodities like coffee (*A. westerdijkiae*, *A. steynii*, *A. ochraceus*) [1], dried fruits (*A. carbonarius*, *A. ochraceus*) [2], spices (*A. ochraceus*) [3] or grapes (*A. carbonarius*) [4,5,6]. On the other hand, the Penicillia are adapted to regions with moderate climate because of their lower temperature growth optimum. *P. verrucosum* can mainly be found on cereals and is responsible for the occurrence of ochratoxin A in cereal-based products [7]. *P. nordicum* often occurs in NaCl rich environments mainly in dry cured meats or cheeses but may even be found in pure NaCl [8,9,10]. Despite the fact that *P. nordicum* is the main ochratoxigenic species in NaCl rich environments, occasionally also *P. verrucosum* can be identified in these commodities [11,12,13]. *P. nordicum* is a very consistent and high ochratoxin A producing species. *A. carbonarius* also produces elevated amounts of ochratoxin A, albeit usually not at the level of *P. nordicum*. In contrast, *P. verrucosum* generally produces only moderate amounts of ochratoxin A. In fact, the production of ochratoxin A by the latter species is highly dependent on the environmental conditions. *P. verrucosum* is also able to produce citrinin, predominantly under conditions of oxidative stress [14], whereas under high NaCl conditions the production of citrinin is shifted towards ochratoxin A [15]. It was recently been shown that the biosynthesis and excretion of ochratoxin A, which carries a chlorine in its molecule, can be regarded as a kind of adaptation to NaCl rich environments. The high production of ochratoxin A under high NaCl conditions by the Penicillia serves as a vehicle which pumps chlorine out of the cell and ensures a certain chlorine homeostasis, which increases the viability of the Penicillia under these conditions [15]. Because of the shift from citrinin towards ochratoxin A in *P. verrucosum* under NaCl conditions, also this species can, under certain circumstances, adapt to this environment and can indeed be found here [11,12,13]. In contrast to the two Penicillia, *A. carbonarius* was never reported to be found in NaCl rich environments. This indicates that *A. carbonarius* is not adapted to NaCl rich habitats, despite the fact that *A. carbonarius* is also able to produce ochratoxin A and should therefore be able to also use the mechanism described above. *A. carbonarius* however occurs in sugar rich environments like grapes, which maybe also osmotically challenging to the fungus.

Changes in the osmolarity of the medium, which can be caused by NaCl or sugars, are usually transmitted to the transcriptional level by the aid of signal cascades. The HOG signal cascade (high osmolarity glycerol) is the most important cascade for this environmental signal. This is a MAP kinase cascade with HOG as the last protein kinase. The phosphorylated form of HOG is the form that subsequently activates the downstream transcription factors. The involvement of this MAP kinase cascade in the regulation of mycotoxin biosynthesis has been shown for trichothecene biosynthesis by *Fusarium graminearum* [16] or alternariol biosynthesis in the case of *Alternaria alternata* [17]. 

Therefore, based on these conditions, the question arose whether the phosphorylation of HOG can be attributed to the regulation of the biosynthesis of ochratoxin A and whether there are differences in the regulation between the three species, which would explain the different adaptation to osmolar challenging conditions. 

## 2. Results

### 2.1. Growth Behavior of *P. nordicum,* *P. verrucosum* and *A. carbonarius* on YES Medium with Increasing Concentrations of NaCl

**Figure 1:**
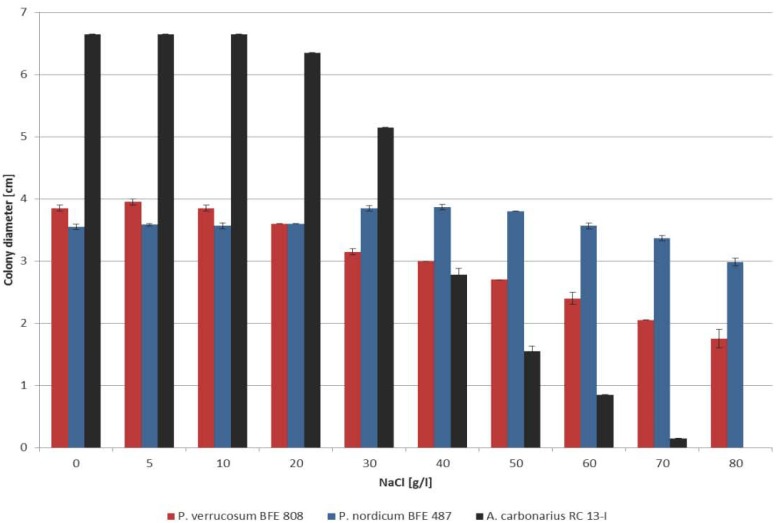
Growth rate of *P. verrucosum, P. nordicum* and *A. carbonarius* after growth on YES medium substituted with increasing amounts of NaCl for five days (*Aspergillus*) and nine days (*Penicillium*) at 25 °C in case of *P. verrucosum* and *P. nordicum* and at 30 °C in case of *A. carbonarius*. An amount of 10 µL of a spore suspension of 10^6^ spores/mL were centrally spotted on an agar plate and incubated. The colony diameters were measured after six and nine days, respectively.

In order to analyze the influence of NaCl on growth and thereby on competitiveness of the three species under increasing NaCl conditions in the YES medium, the diameters of the colony were measured after growth at 25 °C for five days (*Aspergillus*) or for nine days (*Penicillium*) because of the differential growth rates. As can be seen in Figure 1 the colony diameters of the two Penicillia are very similar up to a NaCl concentration of 20 g/L. At higher NaCl concentrations growth of *P. verrucosum* decreased more with increasing NaCl concentrations than growth of *P. nordicum*. In fact at 80 g/L NaCl the colony diameter was less than half of that of the YES medium without NaCl. *P. nordicum* in contrast showed a more adapted growth behavior. This species even showed a growth optimum at 40 g/L NaCl and above this concentration growth only ceases very slowly, reaching 85% of the growth at non-substituted YES medium at the highest NaCl concentration (80 g/L NaCl). In contrast to the Penicillia *A. carbonarius* is much more influenced by increasing NaCl concentrations. Already at 20 g/L NaCl growth is slightly decreased compared to the lower concentrations, however at concentrations beyond this value the growth capability of *A. carbonarius* reduces drastically with further increasing NaCl concentrations. At 80 g/L NaCl no growth at all could yet be identified after an incubation time of five days. 

It has to be kept in mind that only the colony diameter is reported here, which of course is only one parameter for growth. From the morphological point of view the *Penicillium* colonies showed roughly the same morphology (sporulation, color, aerial density of the mycelium) at high NaCl concentrations than the colonies grown at non-modified medium. This was not the case for *A. carbonarius*. At higher NaCl concentrations, this species produced roughly no aerial mycelium and the colony looked very poor indicating the high stress conditions. Taken together these results suggest that the adaptation capacity to high NaCl conditions by the three ochratoxin A producing species reduces from *P. nordicum* to *P. verrucosum* and finally to *A. carbonarius*.

### 2.2. Different Correlations between HOG Phosphorylation and the Onset of Ochratoxin A Biosynthesis in the Three Species

**Figure 2:**
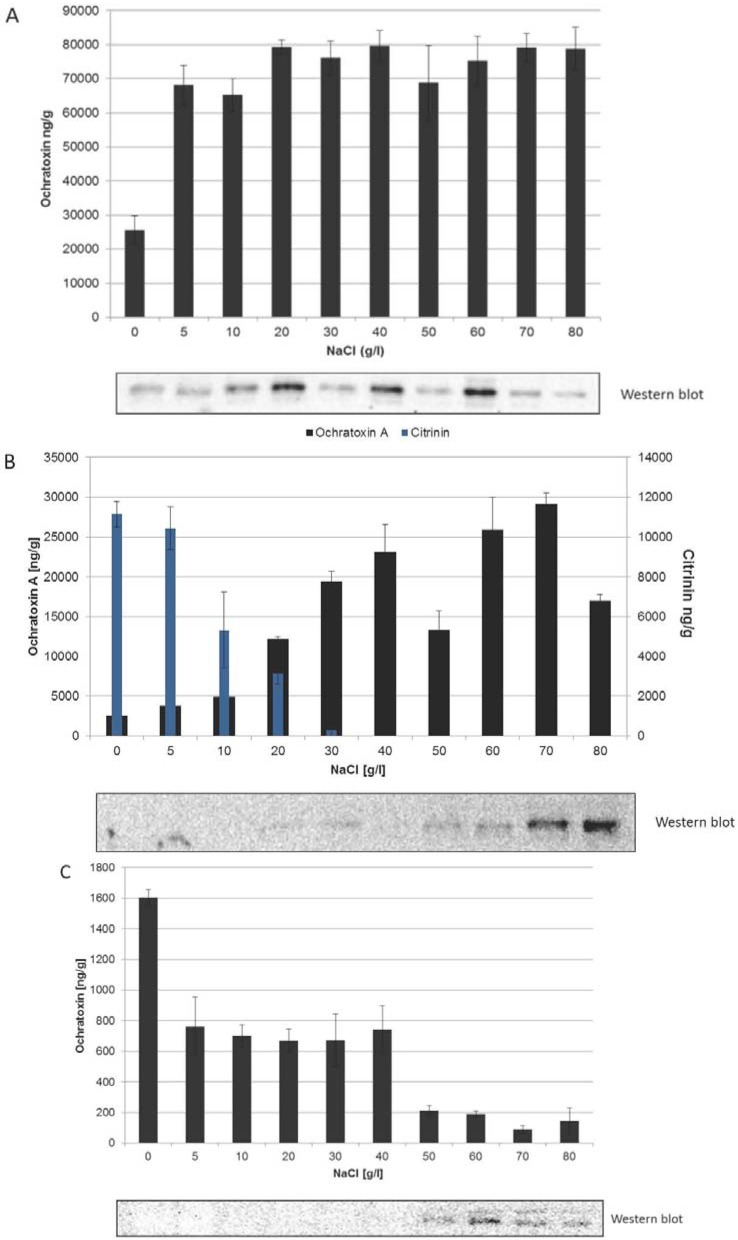
Relation of the production of ochratoxin A and citrinin (in case of *P. verrucosum*) determined by HPLC with the phosphorylation status of the HOG protein, determined by western blotting for *P. nordicum* (**A**); *P. verrucosum* (**B**); and *A. carbonarius* (**C**). The fungal strains were incubated on YES medium with increasing amounts of NaCl at 25 °C (Penicillia) or 30 °C (*Aspergillus*) for nine days. After that time, samples were withdrawn, subjected to HPLC or to western blotting.

Because of a possible relation between NaCl concentration, HOG phosphorylation and regulation of ochratoxin A biosynthesis, the dependence was analyzed in all three ochratoxin A producing species. For this purpose *P. nordicum* BFE487, *P. verrucosum* BFE808 and *A. carbonarius* RC13-I were grown on YES medium with increasing amounts of NaCl. After nine days of incubation at 25 °C and 30 °C respectively, samples were withdrawn. One sample was taken to analyze the kinetics of ochratoxin A biosynthesis by HPLC. The other sample was taken for western blotting to visualize the NaCl dependent phosphorylation of HOG. Figure 2 shows the results. 

The production of ochratoxin A by *P. nordicum* is consistent over a wide concentration range (Figure 2A). Also at high concentrations of 80 g/L NaCl high amounts of ochratoxin were produced. At concentrations below 20 g/L NaCl the amount of ochratoxin produced by this species is reduced. Interestingly the phosphorylation status of HOG follows that of the production of ochratoxin A in that the HOG is phosphorylated over the whole concentration range to a quite constant amount. Also in the control medium, e.g., in unsubstituted YES medium a phosphorylation can be detected and in fact also under these conditions ochratoxin was produced, indicating that YES per se activates the HOG pathway and leads to induction of ochratoxin A biosynthesis. The fact that at some NaCl concentrations (20, 40 and 60 g/L NaCl) HOG exhibits somewhat higher phosphorylation may be attributed to the fact, that the phosphorylation is not constant and quite flexible over time and only a snap shot is shown here. In case of *P. verrucosum* the situation is different (Figure 2B). As mentioned above this species is able to produce, beside ochratoxin A, the structurally very similar mycotoxin citrinin. In this case, as was already shown [15] citrinin is produced on YES medium at low concentrations of NaCl. Beyond 40 g/L NaCl only ochratoxin and no citrinin at all is produced. Interestingly also in the case of *P. verrucosum* BFE808 the phosphorylation status of HOG follows that of ochratoxin A biosynthesis. In contrast to *P. nordicum* only at higher NaCl concentrations a reasonable phosphorylation of HOG becomes apparent. Especially at concentrations above 40 g/L, at conditions where only ochratoxin A and no citrinin is produced by the fungus, the phosphorylation of HOG increases with increasing concentrations of NaCl. 

Taken together the situation in the two Penicillia strongly suggest a relation between HOG phosphorylation and the onset of ochratoxin A biosynthesis, which however is specific for each species.

The situation in the case of *A. carbonarius* is just opposite to that of the Penicillia (Figure 2C). The phosphorylation status is nearly the same as that in *P. verrucosum*. Only at concentrations above 40 g/L a phosphorylation of HOG becomes apparent. In case of *A. carbonarius* a double band arose after western blotting. Because of the fact that the p32 antibodies are usually specific for HOG, the result suggests that multiple forms of HOG exist in *A. carbonarius* (evtl. degradation products). Concerning the phosphorylation status the great difference between *A. carbonarius* and *P. verrucosum* is the fact that exactly at NaCl concentrations which lead to increased HOG phosphorylation the amount of ochratoxin A produced by *A. carbonarius* is drastically reduced. In parallel also the growth of the fungus is strongly inhibited under these conditions. This situation is not only true with *A. carbonarius* RC13-I, which is a moderate to strong ochratoxin A producing strain, but also with *A. carbonarius* ITEM5008, which in our hands is a low producing strain, indicating that this situation is typical for *A. carbonarius* (data with ITEM5008 not shown). The *A. carbonarius* specific results indicate that HOG phosphorylation and ochratoxin A biosynthesis are obviously not coupled in this species in contrast to the situation in the two Penicillia. This means that increasing concentrations of NaCl does not lead to increasing concentrations of ochratoxin A.

### 2.3. Influence of Increasing Amounts of Glucose on Growth, Ochratoxin A Biosynthesis and HOG Phosphorylation by *P. nordicum* and *A. carbonarius*

**Figure 3:**
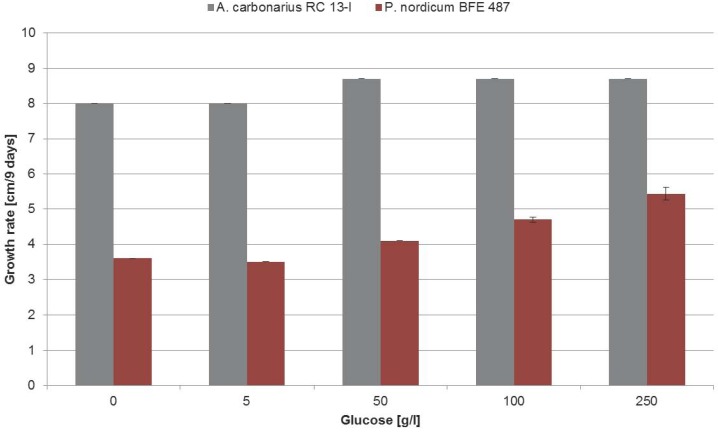
Growth rate of *A. carbonarius* and *P. nordicum* on malt glucose medium (MEA medium). The growth rate was determined as described in legend to Figure 1.

Because it was shown that NaCl as an osmotically active substance, which occurs in the natural habitat of the Penicillia, has a profound but differential effect on the regulation of the biosynthesis of ochratoxin A in the three species, the influence of glucose, a sugar which occurs in the habitat of *A. carbonarius* was analyzed. For this purpose the *A. carbonarius* RC13-I and *P. nordicum* BFE487 were grown on MEA medium with increasing amounts of glucose and growth was measured by determining the colony diameter, ochratoxin A biosynthesis by HPLC and the phosphorylation status of HOG by western blotting. MEA medium instead of YES medium was taken, because YES contains high amounts of sugar (sucrose) which might interfere with the added glucose. The growth behavior of *P. nordicum* and *A. carbonarius* are shown in Figure 3. 

There was a tendency of increasing growth rates with increasing glucose concentrations up to the highest concentration (250 g/L glucose). These results indicate that in contrast to NaCl, high concentrations of glucose are not growth inhibitory to both species. 

**Figure 4:**
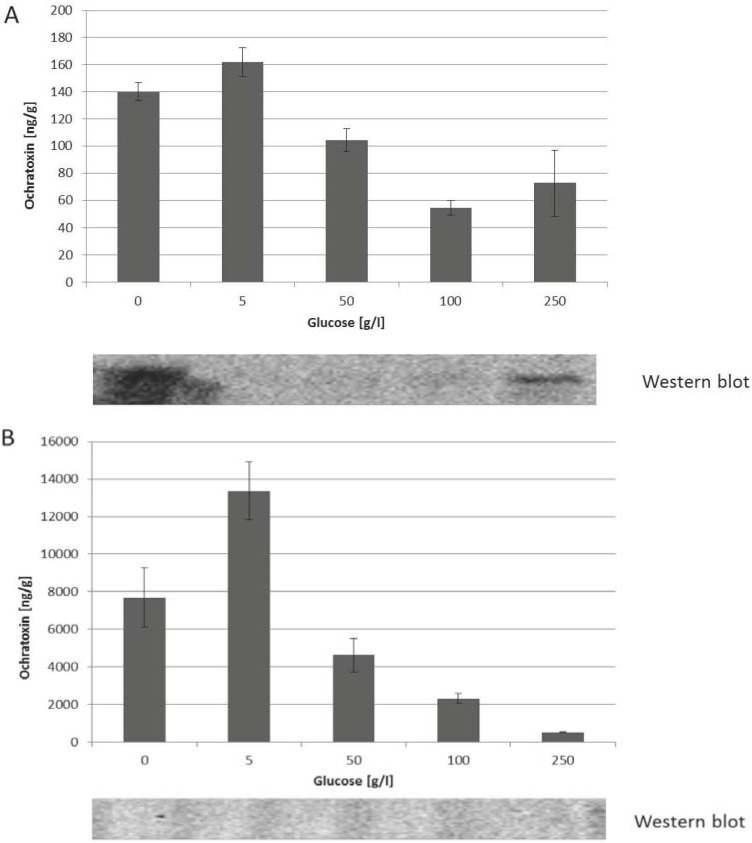
Influence of the glucose concentration on the biosynthesis of ochratoxin A and on the phosphorylation status of HOG by *P. nordicum* (**A**); and *A. carbonarius* (**B**). The fungal strains were grown for nine days on MEA medium with increasing amounts of glucose. After that time, samples were withdrawn and used in HPLC to determine the ochratoxin A produced and for western blot analysis to visualize the phosphorylation status of HOG.

In contrast to the effect on growth, increasing amounts of glucose from 50 to 250 g/L has an inhibitory influence on the biosynthesis of ochratoxin A in the case of *P. nordicum* (Figure 4A) and also in the case of *A. carbonarius* (Figure 4B). 

This decrease is even stronger for *A. carbonarius* than for *P. nordicum*. For both species low concentrations of glucose (5 g/L) lead to an increase in ochratoxin A biosynthesis. 

Interestingly, albeit glucose has an influence on the osmotic status of the medium, it apparently does have only a weak influence on the phosphorylation of HOG. This is in sharp contrast to the situation with NaCl. Under all glucose conditions analyzed no phosphorylation could be observed for *A. carbonarius*. In case of *P. nordicum,* a phosphorylation of HOG could only be observed at the highest glucose concentration (250 g/L). Taken together the results suggest that in contrast to the osmotically active NaCl from the habitat of *Penicillium*, the osmotically active glucose from the habitat of *A. carbonarius* is not an inductor for ochratoxin A biosynthesis for the latter species.

### 2.4. Dependence of the Biosynthesis of Ochratoxin A on Functional HOG

**Figure 5:**
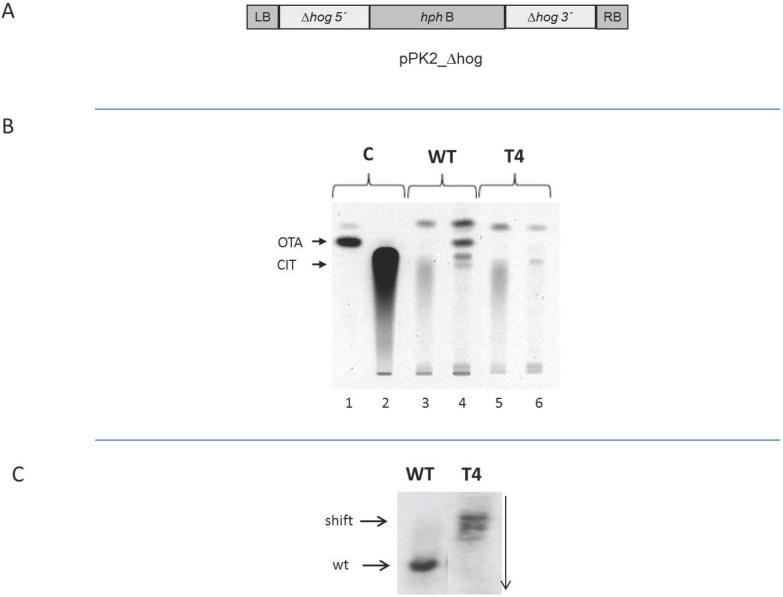
Inactivation of the *hog* gene by ATMT. The cassette used for homologous integration (**A**) into the *hog* gene, was cloned in pPK2 [18]. LB, RB left and right border of the Ti DNA respectively, ∆*hog5*', ∆*hog*3' DNA regions from the upstream (5') and downstream (3') of the *hog* gene, *hph*B hygromycin resisstance gene from *Streptomyces hygroscopicus*. The vector was transformed into *P. verrucosum* BFE808 by *A. tumefaciens* mediated transformation. One of the resulting transformants (T4) was analyzed by TLC to visualize its ochratoxin A and citrinin production capacity (**B**) and to compare it with the wild type (WT). Citrinin shows the typical smear, which is due to the high number of hydroxyl groups which interfere with the silica gel under the conditions used. Transformants with a changed capacity to produce ochratoxin A were further analyzed by Southern blotting (**C**). For Southern blotting the chromosomal DNA of the wild type and the transformant T4 were digested with *Eco*R1, separated on an agarose gel (the vertical arrow indicates the direction of electrophoresis), transferred to a nylon membrane and hybridized to the *hog* gene probe. In the transformant a shift of the hybridization signal towards higher fragment lengths can be seen (**C**) indicating that the vector had integrated into the chromosomal *hog* gene and thereby had inactivated the gene.

It has been demonstrated in the previous experiments that the phosphorylation of the HOG MAP kinase is related to the biosynthesis of ochratoxin in the case of *Penicillium*. In order to analyze whether a functional HOG protein is indeed needed for the production of ochratoxin A under high osmolar conditions, an ATMT (*Agrobacterium tumefaciens* mediated transformation) was carried out with a vector containing the *hyg*B gene between the left and right border of the T-DNA. The *hyg*B gene itself was flanked by fragments of the 5' respectively 3' end of the *P. verrucosum hog* gene (Figure 5A). With this construct an *A. tumefaciens* mediated transformation was carried out. About 10 transformants were obtained. From these 4 transformants were further analyzed, but only the results of one is shown, which was taken for further analysis (transformant 4). The transformant was grown on YES medium (substituted with 80 g/L NaCl to induce ochratotoxin A biosynthesis) respectively MEA medium (MEA medium favors citrinin biosynthesis) containing hygromycin at 25 °C. After 7 days samples were withdrawn and subjected to TLC (Figure 5B). As expected the wild type produced citrinin on MEA medium (Figure 5B, lane 3) and high amounts of ochratoxin on NaCl substituted YES medium (Figure 5B, lane 4). In contrast, the transformant produced no detectable amounts of ochratoxin (Figure 5B, lane 6) but normal amounts of citrinin (Figure 5B, lane 5). A subsequent Southern blot experiment in which a part of the *hog* gene was used as a probe revealed that the vector indeed integrated into the chromosomal *hog* gene, because the hybridization signal shifted to a higher position in the gel (Figure 5C). The visible multiple bands may be due to the homologous integration and further ectopic integrations.

Taken together the results indicate that an inactivation of *hog* abolishes ochratoxin biosynthesis, but not citrinin biosynthesis in *P. verrucosum*, suggesting that ochratoxin biosynthesis is controlled by the *hog* MAP kinase, but not citrinin. 

## 3. Discussion

The three ochratoxigenic species *P. verrucosum*, *P. nordicum* and *A. carbonarius* occur in completely different habitats. *P. nordicum* is adapted to NaCl rich environments like dry cured meats and cheeses [7,8]. *P. verrucosum* can mainly be found in cereals like wheat and is responsible for the production of ochratoxin in the commodity [7] and *A. carbonarius* is the predominant ochratoxigenic species on grapes and grape products [4,6]. *A. carbonarius* and *P. nordicum* do not show any known cross-occurrence, e.g., *P. nordicum* has never been reported to be found on grapes, nor *A. carbonarius* has ever been found on NaCl rich products. In contrast *P. verrucosum* seems to be much more adaptive with respect to the environment. Beside cereals it can occasionally be found also on NaCl rich products like dry cured meats or cheeses [11] or salted olives [12,13] but also occasionally on grapes [19]. It has been discussed earlier that this adaptation to different environments by *P. verrucosum* is paralleled by a shift in the production of secondary metabolites [15]. Under conditions, which impose oxidative stress, like for example under intense light conditions (field conditions), *P. verrucosum* shifts its secondary metabolite profile towards citrinin [14]. Citrinin is supposed to be a light protectant [20] and a potent antioxidant [21] and in fact, in certain cereal samples the contamination with citrinin was more pronounced than with ochratoxin A [22,23]. On the other hand, on substrates with increased concentrations of NaCl, the secondary metabolite profile of *P. verrucosum* is shifted towards ochratoxin A [15]. It has been shown that the production of ochratoxin A under high NaCl conditions is an adaptive reaction [15]. High NaCl conditions not only decrease the *a*_w_ of the substrate, but the chloride ion has additional toxigenic potential, even at *a*_w_ values which have only limited inhibitory effects when caused by other osmolytes [24]. This is also demonstrated in the current work by the drastic reduction in the growth rate of *A. carbonarius* under high NaCl conditions, which was not observed under high glucose conditions, despite the fact that at the highest glucose concentration which did not influence the growth of *A. carbonarius* an *a*_w_ of roughly 0.96 was achieved. About the same *a*_w_ was achieved in the concentration range of 60–70 g/L NaCl which drastically reduces the growth of *A. carbonarius*. Ochratoxin A carries a chlorine in its molecule and under high chloride ion concentrations, which are toxic for the cell [24,25] the permanent production and excretion of ochratoxin A leads to a permanent flow of chlorine out of the cell and ensures at least partial chloride homeostasis. This mechanism lends the ochratoxin A producing fungus a competitive advantage and a higher viability in NaCl rich environments. 

Of course if ochratoxin A biosynthesis is important in NaCl rich environments, the fungus must be able to measure the external salt concentration and relate this to the regulation of ochratoxin A biosynthesis within the cell. Generally, external stimuli like changes in pH, oxidative conditions or changes in the osmotic conditions are transmitted to the genetical level by signal transduction pathways. These signal transduction pathways translate the external signal to the transcriptional level by activating positive or negative transcription factors, which in turn regulates downstream genes. Fungi have various signal cascades, which are activated by different external signals [26]. The signal cascade, which is activated by high osmolarity in the medium, is the *hog* (high osmolarity glycerol) signal cascade [26]. This signal cascade consists of an osmosensor located in the membrane which transmits its signal via a two component system to a set of three consecutive protein kinases (MAP kinases), the last of which is the HOG protein kinase [26]. The phosphorylated form of this kinase is the active form and can activate downstream transcription factors by phosphorylation. 

The involvement of the *hog* signal transduction pathway in the regulation of mycotoxins has been described for trichothecene biosynthesis in case of *F. graminearum* [16], for fumonisin biosynthesis in the case of *F. proliferatum* [27] and for alternariol biosynthesis in *A. alternata* [17]. Interestingly also in the case of *F. graminearum* and *A. alternata* NaCl plays an essential role in the regulation of the respective mycotoxins via HOG. In *P. nordicum* a paralleled activation of HOG phosphorylation and a coincident biosynthesis of ochratoxin A could be observed which suggest dependence between both processes. This dependence could be confirmed by the inactivation of the *hog* gene in *P. verrucosum*. The inactivated transformant was no longer able to produce ochratoxin A under high NaCl conditions in contrast to citrinin biosynthesis, which was not affected. That is in agreement with results from Li *et al.* [28] who showed that citrinin is regulated mainly by another signal cascade, in particular a G-protein/cAMP/PKA signal cascade. 

After several repetitions of these experiments and after being aware that the phosphorylation status of HOG is flexible over time and dependent on the actual conditions, the following general picture could be achieved: *P. nordicum*, which is adapted to high salt and other extreme conditions [9,29] shows a consistent phosphorylation of HOG over all concentrations of NaCl analysed. This is paralleled by a consistently high production of ochratoxin A. The phosphorylation in *P. verrucosum* is not that consistent as in *P. nordicum*. Phosphorylation here occurs only at higher concentrations of NaCl which activates ochratoxin A biosynthesis and thereby represses citrinin biosynthesis. With this mechanism *P. verrucosum* is apparently able to change its adaptation from the cereal to the dry cured or salted food environment. In any case the results strongly suggest that in *Penicillium* the phosphorylation of HOG and the induction of ochratoxin A biosynthesis is coupled, which was also confirmed by gene inactivation in *P. verrucosum*. It has to be kept in mind however that because of the complexity of the regulation and the possible involvement of other regulatory mechanisms the phosphorylation of *hog* is an indication for the induction of the ochratoxin biosynthesis genes, but must not be related to the biosynthesis of ochratoxin A in a one to one ratio. Furthermore, it seems that phosphorylated HOG is not an absolute requirement for ochratoxin A biosynthesis, because ochratoxin A can also be synthesized when HOG phosphorylation is low or non-existing at all (for example under glucose conditions). This indicates that also other factors play a role in the regulation of ochratoxin A biosynthesis. 

The mechanism described above, that the continuous production of ochratoxin A increases the viability of the producing organism in NaCl rich environments should theoretically be due to all ochratoxin A producing organisms. This is however not the case for *A. carbonarius*. Already at concentrations above 40 g/L this species drastically loose the capacity to produce ochratoxin A and concomitantly the ability to grow. According to the results demonstrated here this is at least partly due to the fact that apparently HOG phosphorylation and activation of ochratoxin A biosynthesis is not coupled. As in *P. verrucosum,* HOG is phosphorylated at higher NaCl concentrations, but ochratoxin A biosynthesis in *A. carbonarius* only occurs at lower concentrations of NaCl. Because of this uncoupling between HOG phosphorylation, which is a signal for challenging osmotic conditions, and the activation of ochratoxin A biosynthesis, *A. carbonarius* misses one mechanism to cope with high NaCl stress. 

Because of the fact the *A. carbonarius* is adapted to a sugar rich environment, the influence of high concentrations of glucose on growth and ochratoxin A biosynthesis was analyzed. High concentrations of glucose did not negatively influence the growth of *A. carbonarius* and *P. nordicum*. However high concentration of glucose drastically reduces the production of ochratoxin A, in *A. carbonarius* even more than in *P. nordicum*. Moreover, no glucose dependent phosphorylation of HOG could be observed in *A. carbonarius*. Only at the highest concentration was HOG phosphorylated in *P. nordicum* indicating that glucose induces HOG phosphorylation much less that NaCl in both species. That is in contrast to the situation in *Saccharomyces cerevisiae* [30], suggesting that the influence of glucose in filamentous fungi might be different.

## 4. Experimental Section

### 4.1. Strains and Growth Conditions

*P. nordicum* BFE487 is a strong ochratoxin A producing strain, *P. verrucosum* BFE808 is an ochratoxin A and citrinin producing strain. *A. carbonarius* RC13-I is a consistent but moderately ochratoxin A producing strain, whereas *A. carbonarius* ITEM 5008 only produces low amounts of ochratoxin A in our hands. These strains were used as model strains throughout this study. The strains were routinely grown on malt extract agar (Merck, Darmstadt, Germany) prepared according to the manufacturer’s recommendations, except that 5 g/L glucose were added (MEA). For ochratoxin A biosynthesis the cultures were either incubated on MEA or YES medium (yeast extract 20 g/L; sucrose 150 g/L; agar 20 g/L) which was supplemented with the respective amount of NaCl (0–100 g/L) for 5–7 days at 25 °C (Penicillia) or 30 °C (Aspergilli). 

### 4.2. Growth Assessment

For analyzing the growth rate, the strains were single point inoculated on the agar plates and grown for 9 days under the respective conditions. For this purpose a suspension of 10^6^ spores per mL were prepared by harvesting spores of a seven day old colony with the aid of an inoculation loop and subsequent suspension in TWS solution (25 mL Tween 80 (1% aqueous solution), 8 g NaCl and 1 L distilled water). The spore number were counted in a Thoma chamber and adjusted by adding additional TWS solution if necessary. An amount of 10 µL of that solution was centrally inoculated on an agar plate. After inoculation of the cultures the diameters of the colonies were measured two times rectangular to each other. All experiments were repeated three times. 

### 4.3. Determination of Ochratoxin A and Citrinin by Thin Layer (TLC) and High Pressure Liquid (HPLC) Chromatography

For determination of ochratoxin A/B and citrinin biosynthesis, an agar plug (Ø 1 cm) of the respective colony was taken from the region between center and edge of the colony with the aid of a sterile corer. This agar plug with the adhering mycelium was transferred into 2 mL micro-reaction tubes and 1 mL of chloroform was added. The fungal mycelia were extracted for 30 min at room temperature on a rotary shaker; the mycelia were discarded and the chloroform extract was evaporated to dryness in a vacuum concentrator (Speed Vac, Savant Instruments, Farmingdale, NY, USA). The extracts of the triplicates were merged for quantitative determination of ochratoxin A and citrinin on a Hitachi D-7000 HPLC system (Merck, Tokio, Japan) equipped with an auto-injector, column oven and fluorescence detector. The column oven was set to 40 °C; the fluorescence detector was set to an excitation of 331 nm and an emission of 500 nm. The flow rate was 0.7 mL/min and the injection volume 10 µL. Solvent A consist of 250 mM ortho-phosphoric acid and solvent B of methanol. Separation was carried out on a LiChrospher 100, C18 (250 mm, Ø 4 mm i.d., particle size 5 µm) reversed phase column (VWR International GmbH, Darmstadt, Germany) using the following gradient: 0 min—solvent A 60%, solvent B 40%; 7 min—40%, 60%; 12 min—35%, 65%; 16 min—5%, 95%; 27 min—60%, 40%. The limit of quantification was 25 pg on column. Data collection and handling was done with EZ-Chrome Elite 3.2. All used standards were obtained from Sigma (Taufkirchen, Munich, Germany) with a purity of ≥98%.

### 4.4. Generation and Treatment of Data

Each experiment was repeated at least three times. The mean and SD values for growth and toxin biosynthesis are shown. The western blots gave the same general trends several times.

### 4.5. Protein Extraction

After five days of incubation at 25 °C, the mycelium was scratched off the agar by the aid of a scalpel and 700 mg were transferred directly into 1 mL protein extraction buffer (1 M Tris, 1 M MgCl_2_, 50 mM EDTA, pH 7.5) supplemented with protease inhibitor (cOmplete Mini, EDTA-free Protease Inhibitor Cocktail, Roche, Mannheim, Germany) and phosphatase inhibitor (PhosSTOP, Roche, Mannheim, Germany). The proteins were extracted by repeated sonication for 5 minutes on ice. After spinning for 10 min at 4000 rpm 200 µL trichloracetic acid was added to the separated supernatant protein fraction. The samples were cooled on ice for 30 min and spun for 7 min at 13,000 rpm. The protein pellet was resuspended in 150 µL Laemmli-buffer (0.5 M Tris, 192 mM SDS, 20% glycerol, 10% β-mercaptoethanol and bromphenol blue) and evaporated with ammonia gas to yield deprotonated proteins. The protein extracts were denatured at 95 °C and stored at −80 °C until further analysis.

### 4.6. SDS PAGE and Western Blot

Proteins were separated by SDS PAGE according to Sambrook and Russel [31] and the gel was stained with Coomassie brilliant blue. The relative protein concentration was determined with a Biorad Chemidoc XRS Imaging System (Biorad, Gaithersburg, MD, USA) and the samples were adjusted to equivalent concentrations. The same protein amounts for each sample were loaded onto a new gel and the separated proteins were transferred onto a nylon membrane. The treatment with the p38 antibody specific for phosphorylated HOG (Cell Signaling, Danvers, MA, USA) was performed essentially as described by the manufacturer of the labeling kit. After rinsing and drying, the membrane was incubated with 10 mL LumiGLO solution (Cell Signaling, Danvers, MA, USA). The luminescence was detected with a Biorad Chemidoc XRS Imaging System (Biorad, Gaithersburg, MD, USA). The resulting band intensities after luminescence measuring were evaluated visually.

### 4.7. *A. tumefaciens* Mediated Transformation (ATMT) to Inactivate the *hog* Gene

DNA Fragments covering about 500 bp of the 5' and the 3' end of the *P. verrucosum* *hog* gene were amplified by PCR and cloned upstream and downstream of the hygromycin B resistance gene between the right and left border of the binary vector pPK2 [18]. The resulting disruption cassette is shown in Figure 2A. *A. tumefaciens* AGL-1 cells which carried the vector pPK2 containing the disruption cassette were grown at 25 °C for 48 h in minimal medium which was supplemented with kanamycin (50 μg mL^−1^). The cells were then diluted reaching an OD_660_ of 0.15 in induction medium (IM) (10 mmol/L K_2_HPO_4_, 10 mmol/L KH_2_PO_4_, 2.5 mmol/L NaCl, 2 mmol/L MgSO_4_, 0.7 mmol/L CaCl_2_, 9 mmol/L FeSO_4_, 4 mmol/L (NH_4_)_2_SO_4_, 10 mmol/L glucose, 40 mmol/L 2-[*N*-morpholino] ethan sulfonic acid, pH 5.3, and 0.5% glycerol) in the presence of 200 µmol/L acetosyringone (AS). The cells were further grown for 9 h before mixing with an equal volume of a spore suspension from *P. verrucosum* BFE808. This solution was plated onto cellophane sheets which were placed on the agar plates containing the co-cultivation medium (IM + AS supplemented with 5 mmol/L instead of 10 mmol/L of glucose). After the co-cultivation procedure at 25 °C for 36 h, the cellophane sheets were transferred to M-100 plates (55 mmol/L glucose and 30 mmol/L KNO_3_) plus mineral solution (117 mmol/L KH_2_PO_4_, 28 mmol/L Na_2_SO_4_, 107 mmol/L KCl, 8 mmol/L MgSO_4_ × 7 H_2_O, 9 mmol/L CaCl_2_, 7.8 mmol/L H_3_BO_3_, 5.6 µmol/L MnCl_2_ × 4 H_2_O, 2.3 µmol/L ZnCl_2_, 1.3 µmol/L Na_2_MnO_4_ × 2H_2_O, 2.9 µmol/L FeCl_3_ × 6 H_2_O, and 12.8 µmol/L CuSO_4_ × 5 H_2_O) and 1.5% agar supplemented with hygromycin B (100 μg/mL) as selection medium for fungal transformants and cefoxitin (150 μg/mL) to inhibit the growth of *A. tumefaciens* cells. After incubation for 10 days at 25 °C, the number of hygromycin B-resistant colonies was counted and analyzed further.

### 4.8. Southern Blotting

For Southern blotting experiments the *Eco*RI restriction enzyme digested DNA was separated on a 0.8% agarose gel and transferred onto a nylon membrane filter according to the method of Southern [32]. Hybridization was accomplished under stringent conditions as described by Sambrook and Russel [30]). The 1621 bp *hog* was labeled in a PCR reaction using the primers HOG_Pdigitatum_kpl_for (5'-ATGGCGGAATTCGTGCGTG-3') and HOG_Pdigitatum_kpl_rev (5'-TTATGCGAATGCTTGTCCAGTAGC-3'). The nucleotide mixture contained 11-DIG-UTP. Hybridization and staining of the filter were performed according to the recommendations of the manufacturer of the DIG-UTP Labelling and Detection kit (Boehringer, Mannheim, Germany). 

## 5. Conclusions

Taken together, the ochratoxin A biosynthesis in Penicillia is NaCl concentration dependent and regulated via the phosphorylation of HOG. The constant high production of ochratoxin A by *P. nordicum*, ensured by a consistent phosphorylation status of HOG, renders *P. nordicum* competitive under high salt conditions. The shift from citrinin towards ochratoxin A biosynthesis in *P. verrucosum* under high NaCl conditions, which is also regulated by HOG phosphorylation, make this cereal adapted organism partly competitive on substrates with high NaCl conditions and finally the uncoupling of ochratoxin A biosynthesis and HOG phosphorylation in *A. carbonarius* is apparently one of the reasons why this species is not adapted to NaCl environments. Therefore, one of the ecological reasons for ochratoxin A biosynthesis in *Penicillium* is apparently the maintenance of chloride homeostasis [15]. This is not the case in *A. carbonarius*. The ecological reason for the production of ochratoxin A by *A. carbonarius* must be different. Glucose (or other sugars) as a typical osmolytic active constituent of its natural habitat only has a negative influence of the biosynthesis of ochratoxin A. Therefore, a reasonable ecological reason for the biosynthesis of ochratoxin A by *A. carbonarius* cannot yet be deduced. 

## Abbreviations

YES: yeast extract sucrose medium
MEA: malt extract agar
HOG signal pathway: high osmolarity glycerol signal pathway
MAPK: mitogen activated protein kinase
ATMT: *Agrobacterium tumefaciens* mediated transformation
TLC: thin layer chromatography
